# Homeodomain Protein Scr Regulates the Transcription of Genes Involved in Juvenile Hormone Biosynthesis in the Silkworm

**DOI:** 10.3390/ijms161125945

**Published:** 2015-11-02

**Authors:** Meng Meng, Chun Liu, Jian Peng, Wenliang Qian, Heying Qian, Ling Tian, Jiarui Li, Dandan Dai, Anying Xu, Sheng Li, Qingyou Xia, Daojun Cheng

**Affiliations:** 1 State Key Laboratory of Silkworm Genome Biology, Southwest University, Chongqing 400715, China; keven190@sina.com (M.M.); mlliuchun@163.com (C.L.); theppone@163.com (J.P.); wenliang20081103@126.com (W.Q.); ljr1994@email.swu.edu.cn (J.L.); dai_dandan91@163.com (D.D.); 2 The Sericultural Research Institute, Jiangsu University of Science and Technology, Jiangsu 212018, China; qianheying123@163.com (H.Q.); srixay@126.com (A.X.); 3 The Sericultural Research Institute, Chinese Academy of Agricultural Sciences, Jiangsu 212018, China; 4 Institute of Plant Physiology and Ecology, Shanghai Institutes for Biological Sciences, Chinese Academy of Sciences, Shanghai 200032, China; tianling@sibs.ac.cn (L.T.); shengli@sippe.ac.cn (S.L.)

**Keywords:** silkworm, sex combs reduced, juvenile hormone, biosynthesis, transcriptional regulation

## Abstract

The silkworm *Dominant trimolting* (*Moltinism*, *M^3^*) mutant undergoes three larval molts and exhibits precocious metamorphosis. In this study, we found that compared with the wild-type (WT) that undergoes four larval molts, both the juvenile hormone (JH) concentration and the expression of the JH-responsive gene *Krüppel homolog 1* (*Kr-h1*) began to be greater in the second instar of the *M^3^* mutant. A positional cloning analysis revealed that only the homeodomain transcription factor gene *Sex combs reduced* (*Scr*) is located in the genomic region that is tightly linked to the *M^3^* locus. The expression level of the *Scr* gene in the brain-corpora cardiaca-corpora allata (Br-CC-CA) complex, which controls the synthesis of JH, was very low in the final larval instar of both the *M^3^* and WT larvae, and exhibited a positive correlation with JH titer changes. Importantly, luciferase reporter analysis and electrophoretic mobility shift assay (EMSA) demonstrated that the Scr protein could promote the transcription of genes involved in JH biosynthesis by directly binding to the *cis*-regulatory elements (CREs) of homeodomain protein on their promoters. These results conclude that the homeodomain protein Scr is transcriptionally involved in the regulation of JH biosynthesis in the silkworm.

## 1. Introduction

Insect larvae generally undergo several molting events before larval-pupal metamorphosis and these molting processes divide the entire larval period into separate stages, which are called instars [1,2,3]. The number of larval molts varies across insect taxa, from two in the fruit fly (*Drosophila melanogaster*) to 33 in the mayfly (*Leptophlebia cupida*) [4]. Even within a specific species, such as the silkworm (*Bombyx mori*), variants of the numbers of larval molts also exist [5,6]. This variation in the larval molt number across insects is referred to by the term moltinism, and insect species with different moltinisms can be applied to decipher the genetic basis of larval molting.

Insect larval molting and metamorphosis are orchestrated by two types of endocrine hormones, juvenile hormone (JH) and ecdysone [7]. JH is produced in the corpora allata (CA) and JH biosynthesis is modulated by two neuropeptides from the brain, allatotropin (AT) and allatostatin (AST) [8]. Ecdysone is synthesized in the prothoracic gland (PG) after induction by neuropeptide prothoracicotropic hormone (PTTH) from the brain [9]. In each larval instar, high titers of JH during the early stages maintain larval growth, whereas the presence of an elevated ecdysone pulse during the late stage initiates larval-larval molting. In the final larval instar, the disappearance of JH elicits pupal commitment, then a dramatically elevated ecdysone pulse triggers larval-pupal metamorphosis [10].

The silkworm is an economically important insect and has been well studied from the genetic, biochemical, and genomic perspectives [5,11]. Generally, silkworm larvae undergo four molts and form five instars. Interestingly, several moltinism varieties have been discovered in silkworms, including a recessive *nonmolting* (*nm*) variety with no larval molting, a recessive *dimolting* (*mod*) variety with two larval molts, and *recessive trimolting* (*rt*) and *dominant trimolting* (*Moltinism*, *M^3^*) varieties with three larval molts. Recently, a genome-based positional cloning approach was successively used to identify genes that are closely linked with several silkworm moltinism mutations, including the short-chain dehydrogenase/reductase gene *shroud* (*sro*) for the *nonmolting-glossy* (*nm-g*) mutant and the cytochrome P450 gene *CYP15C1* for the *mod* mutant [6,12]. A 5.2 kb insertion in the third exon of the *sro* gene leads to a deficiency in ecdysone biosynthesis in the silkworm *nm-g* mutant [12], and a 68 bp deletion in the *CYP15C1* gene results in a lack of JH biosynthesis in the *mod* mutant [6]. The currently available evidence clearly indicates that silkworm moltinism mutations are always associated with disruption of the biosynthesis pathway of either JH or ecdysone.

To date, the genetic basis of the silkworm *M^3^* mutant remains poorly understood.Previous genetic studies have reported that the locus of the silkworm *M^3^* mutant is located at the 24.1 cM locus on chromosome 6, which includes a homeodomain gene cluster [13]. In this work, we observed that compared with the wild-type (WT) silkworm, which undergoes four larval molts, the greater concentration of JH titer in the *M^3^* mutant at the beginning of the second larval instar most probably cause the trimolting phenotype. Positional cloning analysis identified that the *Sex combs reduced* (*Scr*) gene, a member of the homeodomain gene family, is located at the *M^3^* locus. Further analysis demonstrated that the Scr protein regulates the transcription of several genes that are involved in JH biosynthesis.

## 2. Results

### 2.1. The Silkworm M^3^ Mutant Larvae Exhibits an Enhancement of JH Biosynthesis in the Second Instar

**Figure 1:**
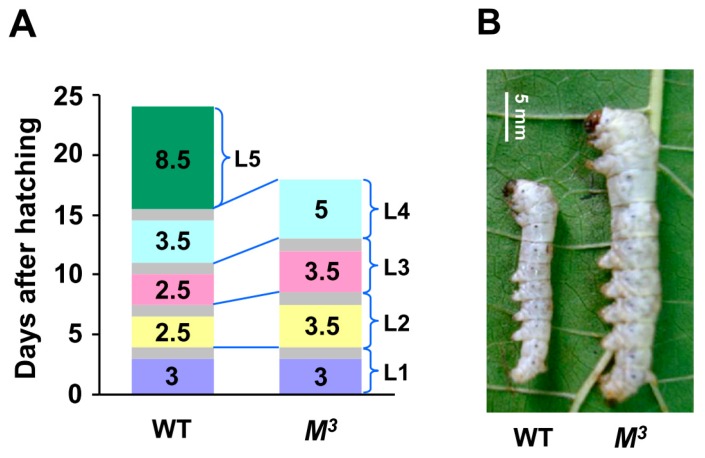

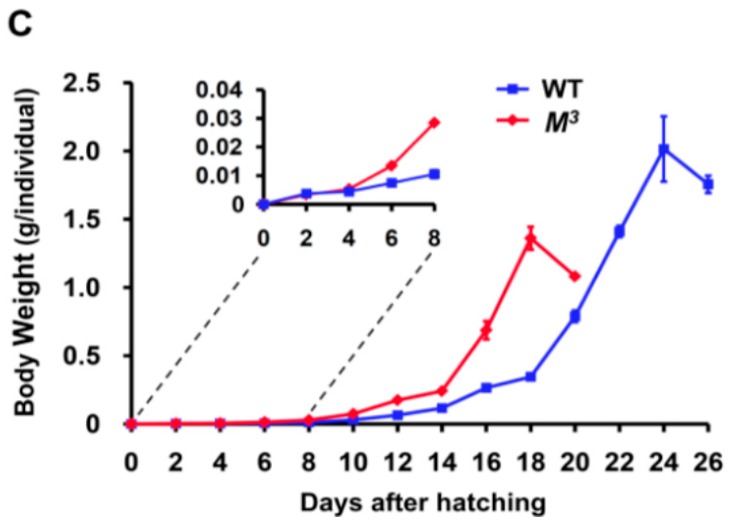
Developmental phenotypes of the *M^3^* mutant. (**A**) Precocious larval-pupal metamorphosis in the *M^3^* mutant. The *M^3^* larvae undergo only three molts. The feeding duration of the second and third instars is greater than that of the same instars in the WT larvae, and the entire larval period of the *M^3^* mutant is six days shorter than that of WT. The gray areas denote the process of larval molting. L, larval instar; L1 to L5 respectively represents the first to the fifth larval instar; (**B**) Morphology of the *M^3^* and WT larvae on the 11th day after hatching. The WT larvae have progressed into the third larval molting, but the *M*^3^ larvae remain at day two of the third larval instar, and their body size is bigger than that of the WT larvae; (**C**) Comparison of the body weights of the *M^3^* and WT larvae. We randomly chose a certain number of both *M^3^* and WT individuals every 48 h from the first day they were hatched and got the average body weight. Beginning with the second instar, the body weight of the *M^3^* larvae increased faster than that of the WT larvae. Because of the lack of a larval instar, the final body weight of the *M^3^* larvae was less than that of the WT larvae. The error bars represent the mean ± S.E. (*n* = 3).

The silkworm *M^3^* strain is a dominant trimolting mutant. Compared with the WT larvae, which initiated metamorphosis in the fifth instar after four rounds of larval molting, the *M^3^* larvae underwent a precocious metamorphosis in the fourth instar, and the entire larval duration was shortened by approximately six days (Figure 1A). Importantly, beginning with the second instar, the developmental progression of the *M^3^* and WT larvae revealed an obvious difference. The initiation of the second molting in the *M^3^* larvae occurred approximately 24 h later than in the WT larvae, and the duration of the third and the fourth instars in the *M^3^* larvae was also prolonged (Figure 1A). Consequently, the body size and body weight of the *M^3^* larvae were greater than those of the WT larvae during the former four instars. Morphologically, at the 11^th^ day after hatching, the WT larvae had grown into the third larval molting, whereas the *M^3^* larvae remained at day two of the third larval instar (Figure 1A), but their body size was bigger than that of the WT larvae (Figure 1B). Statistically, the body weight of the *M*^3^ larvae at the end of the fourth instar was almost equal to that of the WT larvae in the late fifth instar (Figure 1C). Because an appropriate body weight is critical for initiating metamorphosis [14,15], we propose that the early attainment of the critical weight in the *M^3^* larvae may result in its precocious metamorphosis.

**Figure 2:**
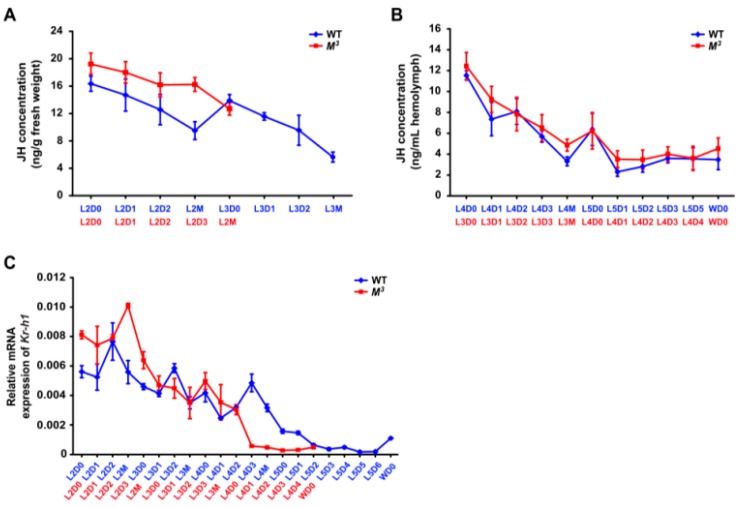
JH concentration and developmental expression of the *Kr-h1* gene in the *M^3^* and WT larvae. (**A**) JH concentration in the whole bodies of the *M^3^* and WT larvae during early instars. GC-MS analysis indicated that the JH titer in the whole body of the *M^3^* mutant larvae was higher than that of the WT larvae in the second instar; (**B**) JH concentration in the hemolymph of the *M^3^* and WT larvae during late instars. GC-MS analysis indicated that the JH titer in the hemolymph of either the *M^3^* mutant or WT larvae were similarly decreased to low levels at the final instar; (**C**) *Kr-h1* expression in the integument of the *M^3^* and WT larvae. qRT-PCR analysis indicated that the expression level of the JH-responsive gene *Kr-h1* was higher in the *M^3^* larvae than in the WT larvae in the second instar, and *Kr-h1* was expressed at very low levels in the final instar of both the *M^3^* and WT larvae. L, larval instar; L2 to L5 respectively represents the second to the fifth larval instar; D, day; D0, initial day; D1 to D6 respectively represents the first to the sixth day of a larval instar; M, molting; W, wandering. The error bars represent the mean ± S.E. (*n* = 3).

Given that larval molting and metamorphosis in insects are controlled by JH and ecdysone [7], we speculated that the precocious metamorphosis in the *M^3^* mutant was likely caused by a disruption of the biosynthesis or signaling of either JH or ecdysone. To test this hypothesis, we first compared the changes in the JH titers during the development of the *M^3^* and WT larvae. Gas chromatography-mass spectrometry (GC-MS) analysis revealed that the JH titers gradually decreased from the second to the last instar in both the *M^3^* and WT larvae, and it was high at the beginning of each instar, and then decreased to a low level (Figure 2A,B). Intriguingly, we found that the absolute concentration of JH titer in the *M^3^* larvae was greater than that in the WT larvae in the second instar (Figure 2A). In addition, a quantitative RT-PCR (qRT-PCR) assay demonstrated that in the integument, which is a target tissue of JH action, the expression of the *Krüppel homolog 1* (*Kr-h1*) gene, which is transcriptionally regulated by JH and then involved in JH signaling [16], was markedly higher in the *M^3^* larvae than in the WT larvae in the second instar (Figure 2C). Moreover, the *Kr-h1* expression also decreased to very low levels in both the *M^3^* and WT larvae in the last instar (Figure 2C). These expression profiles of *Kr-h1* in the integument are consistent with the typical developmental changes in the JH titers in both the *M^3^* and WT larvae (Figure 2A,B), indicating that the phenotype of the *M^3^* mutant may be caused by the enhancement of JH biosynthesis in the second instar.

We further examined the biosynthesis and signaling of ecdysone in both the *M^3^* and WT larvae. Radioimmunoassay (RIA) data revealed that the developmental changes in the ecdysone titers were similar for the *M^3^* and WT larvae from the second instar to the final instar, and dramatically increased at the beginning of wandering (Figure S1A,B). In addition, the expression of the ecdysone receptor (*EcR*) gene involved in ecdysone signaling was increased to similar levels before each molting and at the beginning of wandering in the *M^3^* and WT larvae (Figure S1C). Furthermore, the *EcR* expression level exhibited no difference in the second instar between the *M^3^* and WT larvae (Figure S1C), thereby suggesting that the ecdysone pathway is not closely linked to the *M^3^* mutant.

### 2.2. JH Analogue Treatment Postpones Larval Molting and Induces Trimolting Phenotype

**Figure 3:**
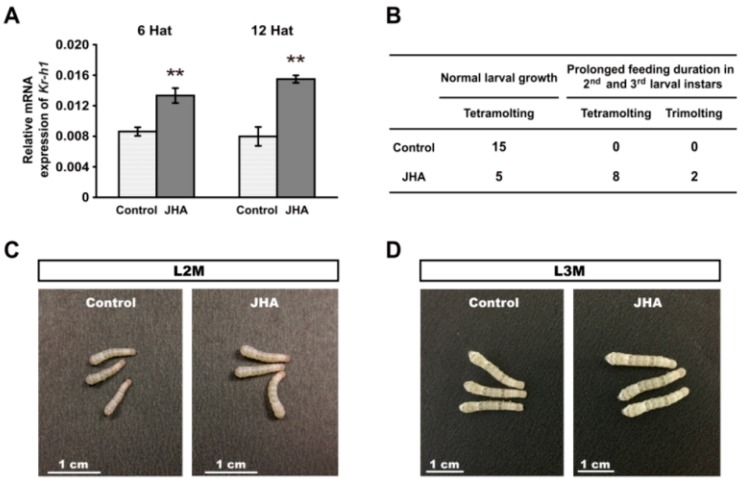

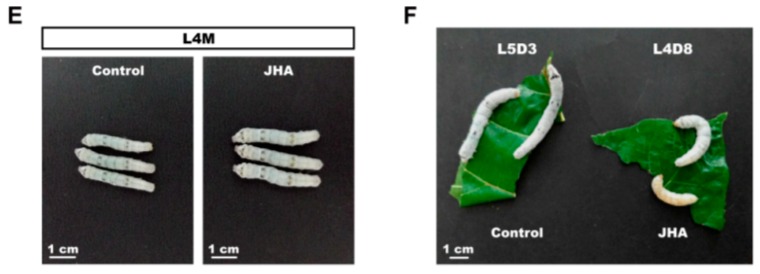
Change of larval growth and molting by JHA application at the beginning of the second instar. (**A**) The expression of *Kr-h1* was increased at 6 h and 12 h after JHA treatment. The error bars represent the mean ± S.E. (*n* = 3). The significance of the difference between data sets was calculated using two-tailed Student’s *t*-test. ** *p* < 0.01, compared with the control. Hat, hours after treatment; (**B**) The effects of JHA application on larval growth and development; (**C**–**E**) Morphology of the JHA-treated and control larvae at the second , third, and fourth larval molting. The body size of the JHA-treated larvae is bigger than that of the control larvae; (**F**) Morphology of the JHA-application-induced trimolting larvae and the control tetramolting larvae at the same development point in their final instars. When control larvae develop to day three of the fifth instar, the JHA-treated larvae have initiated wandering although they remain at day eight of the fourth instar. L, larval instar; L2 to L5 respectively represents the second to the fifth larval instar; D, day; D3, the third day of a larval instar; D8, the eighth day of a larval instar; M, molting.

Because the enhancement of JH biosynthesis at the beginning of the second larval instar is closely linked to the silkworm *M^3^* mutant, we tested whether the treatment of JH analogue (JHA) methoprene on the newly molted second instar larvae of WT with four molts will result in developmental changes that are similar to the *M^3^* mutant. qRT-PCR analysis showed that the expression of the JH-responsive gene *Kr-h1*was significantly increased in the integument at 6 h and 12 h after JHA treatment (Figure 3A). As expected, we observed that, compared with the control larvae, the feeding duration of either the second or the third larval instar of 10 individuals of 15 silkworm larvae with JHA treatment were prolonged for about 12 h, and their body size was also increased before larval molting (Figure 3B–E). Intriguingly, two JHA treated larvae only underwent three larval molts and initiated wandering at the eighth day after the third larval molting when the control larvae were in the middle of the fifth instar (Figure 3B,F), confirming that JHA application at the beginning of the newly molted second instar larvae could induce trimolting. Therefore, these results strongly supported that the trimolting phenotype of the *M^3^* mutant may be caused by the elevation of JH titer at the beginning of the second instar.

### 2.3. Positional Cloning Maps Scr Gene at the M^3^ Locus

**Figure 4:**
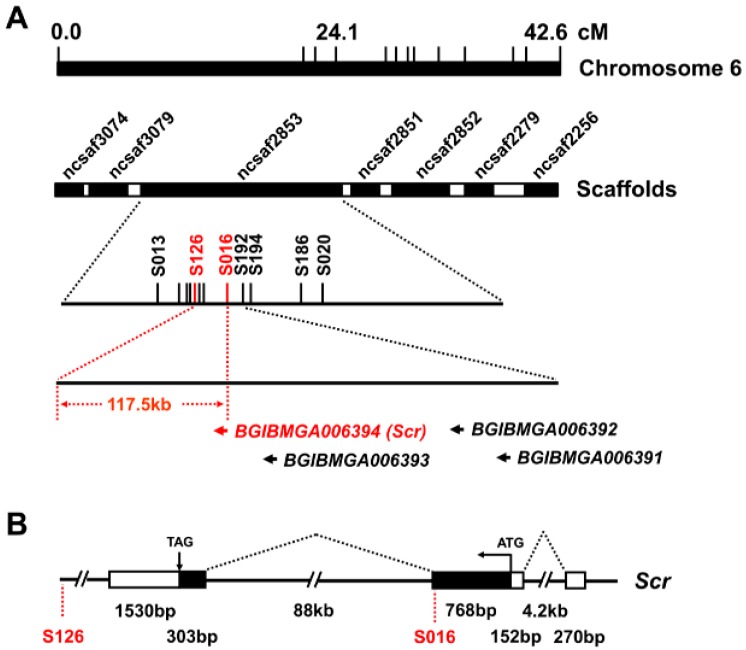

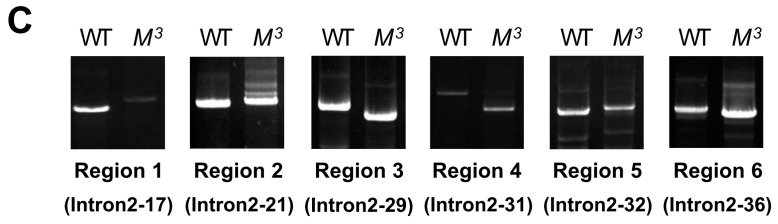
Positional cloning of the *M^3^* locus. (**A**) Positional mapping of the silkworm *M^3^* locus on chromosome 6. Using SNP markers on chromosome 6 and 1307 BC1 trimolting individuals, the region linked to the *M^3^* locus could be narrowed down to the 117.5 kb genomic region between SNP markers S126 and S016 on the scaffold of nscaf2853. Only one predicted gene, *BGIBMGA006394*, was located in this region, and this gene is annotated as *Scr*; (**B**) Schematic structure of the *Scr* gene. By searching the silkworm genome databases SilkDB and KAIKObase, the full-length cDNA sequence of the candidate *Scr* gene was determined to be comprised of three exons and two introns, and the second intron and the third exon are located in the genomic region that is linked to the *M^3^* locus. The black boxes, white boxes, and dotted diagonal lines indicate coding sequences, UTRs, and introns, respectively; (**C**) Genomic differences in intron 2 of the *Scr* gene between the *M^3^* and WT strains. Genomic PCR analysis confirmed that six regions in intron 2 of the *Scr* gene exhibited sequence variations of greater than 100 bp in length between the *M^3^* and WT strains. The names of the primers used for amplifying these regions are presented in parentheses.

To identify the genes located at the *M^3^* locus, we conducted positional cloning using 1307 trimolting individuals from the progeny of 37 pairs of backcross 1 (BC1) by crossing WT females and F1 heterozygote males obtained from the crossing of the *M^3^* mutant and WT strains. Using single nucleotide polymorphism (SNP) markers from both a previous report [17] and our newly developed silkworm genetic variation map [18], as listed in Table S1, we mapped the *M^3^* locus within the 117.5 kb genomic region between the SNP markers S126 and S016 on the Bm_nscaf2853 of chromosome 6 (Figure 4A). This region contained only the downstream sequence of the second exon of the predicted gene, *BGIBMGA006394* (*BMgn006394*/*Gene03398* in the KAIKObase) (Figure 4B), which encodes the Sex combs reduced (Scr) protein that belongs to the homeodomain transcription factor family.

We compared the sequences of the *Scr* gene from the *M^3^* mutant and WT strains. First, a coding sequence (CDS) analysis indicated that, except for a mutation of the SNP marker S016 that resulted in a change from Ser at position 224 to Asn, no other sequence differences between the *M^3^* and WT strains were identified in CDS regions of the *Scr* genes (Figure S2A,B). Next, an examination of the 3′ untranslated regions (UTRs) indicated that, although some sequence variations existed in the 3′ UTR of the *Scr* genes from *M^3^* and WT strains, these variations were also present in other trimolting silkworm strains (Figure S2C), thereby indicating that these variations could not contribute to the *M^3^* mutant. Finally, given that intron 2 of the *Scr* gene is located in the *M^3^* linked region and is 88 kb in length (Figure 4B), we further determined whether sequence variations existed in intron 2 of the *Scr* genes between the *M^3^* and WT strains. Using 39 pairs of specific primers that covered the genomic regions of intron 2 (Table S1), we obtained different genomic regions of intron 2 via genomic PCR experiments. Electrophoresis and sequencing of the PCR products revealed that at least six regions exhibited a sequence variation of greater than 100 bp in length (Figure 4C and Table S2). By searching the National Center for Biotechnology Information (NCBI) database using the Basic Local Alignment Search Tool for nucleotide sequences (BLASTn), we observed that the insertions in both regions 1 and 5 and the deletion in region 3 were homologous to a retrotransposon, the mariner-like transposable element 1 of *Bombyx mandarina* (Bm1), thereby suggesting that the variations in intron 2 may contribute to the roles of the *Scr* gene in the *M^3^* mutant.

### 2.4. Scr Gene is Highly Expressed in Br-CC-CA during Early Larval Stages

**Figure 5:**
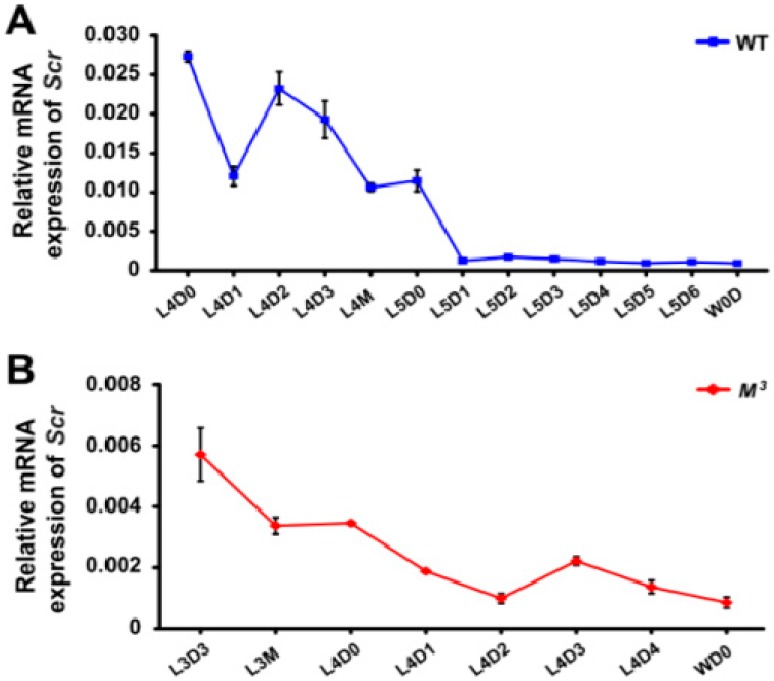

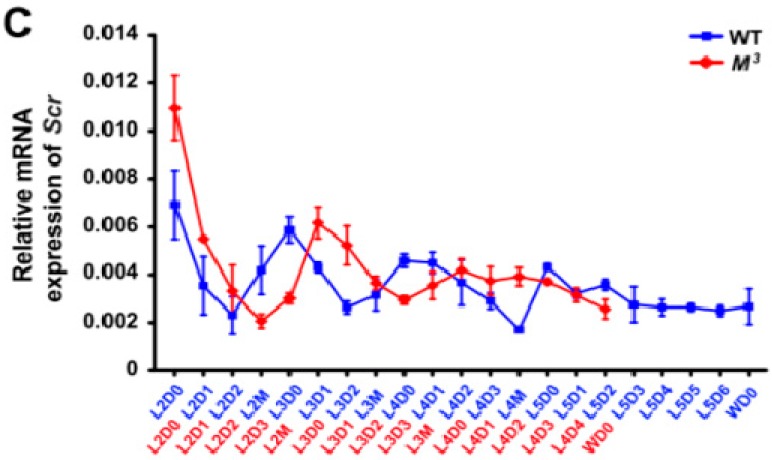
Developmental expression of the *Scr* gene in the *M^3^* and WT larvae. (**A**,**B**) qRT-PCR analysis of *Scr* expression in the Br-CC-CA complex. The results indicated that *Scr* was highly expressed in the Br-CC-CA complex in the early stages of either the fourth instar of WT larvae or the third instar of *M^3^* larvae, and that it was dramatically decreased in the final instar. This expression of the *Scr* gene exhibited a close correlation with the JH titer changes during the same stages; (**C**) qRT-PCR analysis of *Scr* expression in the areas of the head that mainly contain the Br-CC-CA complex. The results confirmed that the *Scr* expression levels in the head were higher in the *M^3^* larvae than in the WT larvae during the early feeding stage of the second instar, and these levels exhibited a positive correlation with the difference in the JH titers between the *M^3^* and WT larvae. L, larval instar; L2 to L5 respectively represents the second to the fifth larval instar; D, day; D0, initial day; D1 to D6 respectively represents the first to the sixth day of a larval instar; M, molting; W, wandering. The error bars represent the mean ± S.E. (*n* = 3).

Insect larval molting is precisely regulated by JH and ecdysone. To explore the potential crosstalk between Scr and the biosynthesis of either JH or ecdysone, we profiled the temporal expression of the *Scr* gene in both the brain-corpora cardiaca-corpora allata (Br-CC-CA) complex, which controls the synthesis of JH, and the PG, where ecdysone is synthesized. qRT-PCR examination indicated that in the Br-CC-CA complex of the WT larvae during the last two instars, *Scr* expression was high during the early feeding stages of the fourth instar, subsequently decreased before the fourth molting, and became very low in the final instar (Figure 5A). This changing tendency of *Scr* expression was similar to that observed during the final two instars of the *M^3^* mutant (Figure 5B), and was positively correlated with the changes in the JH titer (Figure 2B). In addition, because it was somewhat difficult to isolate the Br-CC-CA complex at the early larval stages from the second to third larval instars, we further analyzed the developmental expressions of the *Scr* gene in areas of the head that mainly contain the Br-CC-CA complex. Our results demonstrated that *Scr* expression in the head was high at the beginning of each larval instar and decreased before larval molting in both the *M^3^* and WT strains (Figure 5C), which exhibited a positive correlation with the JH titer change (Figure 2A,B). Notably, *Scr* expression was higher in the *M^3^* strain than that in the WT strain during the early feeding stage of the second instar (Figure 5C), which is consistent with the greater concentration of JH titer and the higher expression level of *Kr-h1* in the *M^3^* mutant (Figure 2A,C). These data indicate that the *Scr* gene might be involved in JH biosynthesis.

We further investigated the developmental expression of the *Scr* gene in the PG. As shown in Figure S3, although *Scr* was highly expressed in the PG during the final molting in both the *M^3^* and WT larvae, its expression was very low during the final instar and before the larval-pupal metamorphosis, which does not correspond to the dramatic elevation of the ecdysone titer at the beginning of wandering (Figure S1B). Taken together, our results suggest that the *Scr* gene is involved in JH biosynthesis but does not contribute to ecdysone biosynthesis.

### 2.5. Scr Protein Regulates the Transcription of Genes Involved in JH Biosynthesis

**Figure 6:**
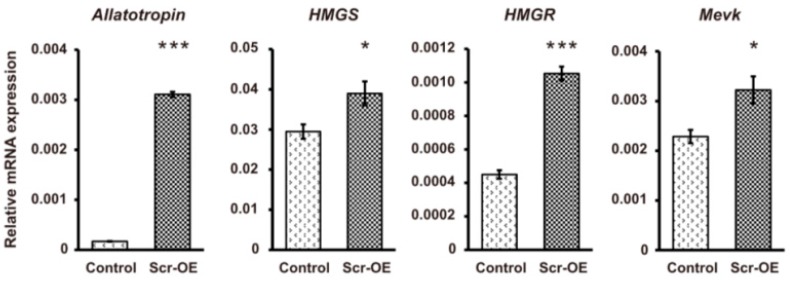

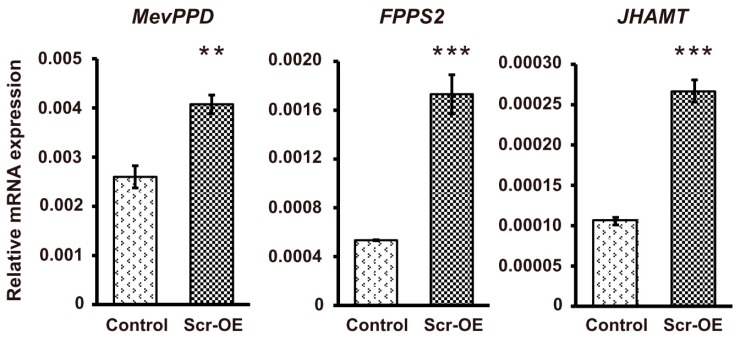
The effects of *Scr* overexpression on the transcription of genes involved in JH biosynthesis. The vector used to overexpress the silkworm *Scr* gene was transfected into BmE cells. The *EGFP*-overexpressing vector was used as a control. The cells were collected 72 h after transfection for qRT-PCR analysis. Among the genes involved in JH biosynthesis, the transcription of the *Allatotropin* gene and the genes that encode key enzymes, including *HMGS*, *HMGR*, *MevK*, *MevPPD*, *FPPS2*, and *JHAMT*, were significantly induced after *Scr* overexpression. OE, overexpression. The error bars represent the mean ± S.E. (*n* = 3). The significance of the difference between data sets was calculated using two-tailed Student’s *t*-test. * *p* < 0.05; ** *p* < 0.01; *** *p* < 0.001, compared with the control.

To investigate whether the Scr protein is involved in regulating the transcription of genes that control JH biosynthesis, we transfected the *Scr* overexpression vector 1180-*hr3*-*A4*/*Flag*-*Scr* or the *enhanced green fluorescent protein* (*EGFP*) overexpression vector 1180-*hr3*-*A4*/*EGFP*, which was used as a negative control, into the silkworm embryo-derived (BmE) cells. Western blotting showed that the Scr protein was successfully overexpressed (Figure S4). Previous studies have reported that insect JH biosynthesis involves multiple enzymatic steps and is modulated by several neuropeptides [7,19,20,21]. A qRT-PCR analysis indicated that the transcription of the gene that encodes the neuropeptide allatotropin as a stimulator of CA activity and the genes that encode key enzymes involved in JH biosynthesis, such as *HMGS*, *HMGR*, *MevK*, *MevPPD*, *FPPS2*, and *JHAMT*, could be markedly increased after *Scr* overexpression (Figure 6). In contrast, the transcription of the genes involved in ecdysone biosynthesis, including *PTTH*, *PTSP*, *Phantom*, and *Shadow*, was not altered following *Scr* overexpression (Figure S5). These results further suggest that Scr plays important roles in regulating the transcription of genes that control JH biosynthesis, but is not involved in the transcriptional regulation of ecdysone biosynthesis-related genes.

**Figure 7:**
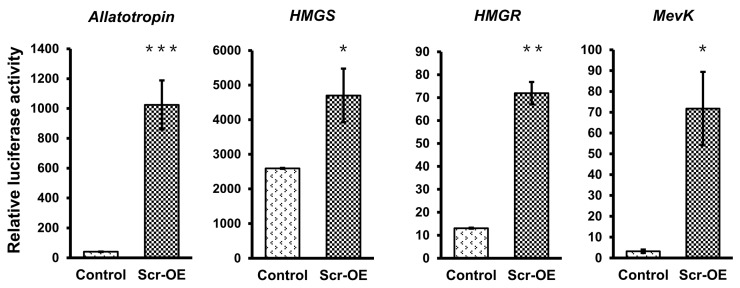

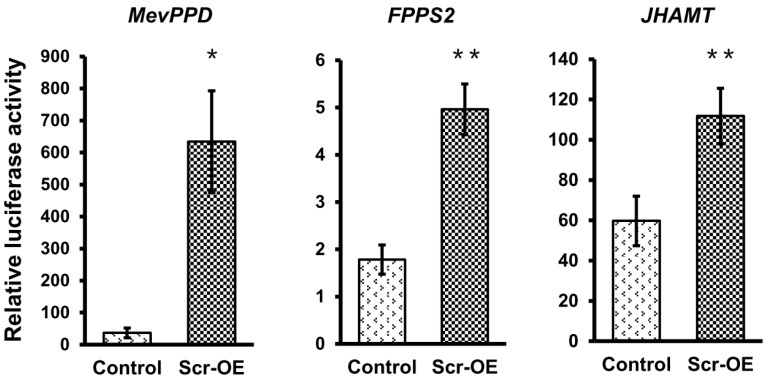
The effects of *Scr* overexpression on the activities of the promoters of genes involved in JH biosynthesis. Luciferase reporter analysis in BmE cells indicated that the activities of the promoters of seven genes whose transcription was induced after *Scr* overexpression, including *Allatotropin*, *HMGS*, *HMGR*, *MevK*, *MevPPD*, *FPPS2*, and *JHAMT*, were also up-regulated by *Scr* overexpression. OE, overexpression. The error bars represent the mean ± S.E. (*n* = 3). The significance of the difference between data sets was calculated using two-tailed Student’s *t*-test. * *p* < 0.05; ** *p* < 0.01; *** *p* < 0.001, compared with the control.

To further confirm whether the transcription of these JH biosynthesis-related genes can be regulated by Scr, we used the online MatInspector program (http://www.genomatix.de/) and predicted several potential *cis*-regulatory elements (CREs) for homeodomain transcription factors on the promoter regions that are approximate 2 kb in length upstream of the ATG start codons of these selected genes (Figure S6). According to this result, we next designed the promoter-specific primers listed in Table S1 to clone these promoter sequences into the pGL3-basic vector and then respectively co-transfected each of the recombinant pGL3 vectors with the *Scr* overexpression vector or the *EGFP* overexpression vector into BmE cells. The luciferase reporter assay indicated that *Scr* overexpression significantly increased the activities of the promoters of these JH biosynthesis-related genes (Figure 7). These results strongly support the notion that the Scr protein regulates the transcription of genes in the JH biosynthesis pathway of the silkworm.

### 2.6. Scr Protein Directly Binds to the Promoters of Genes Involved in JH Biosynthesis

**Figure 8:**
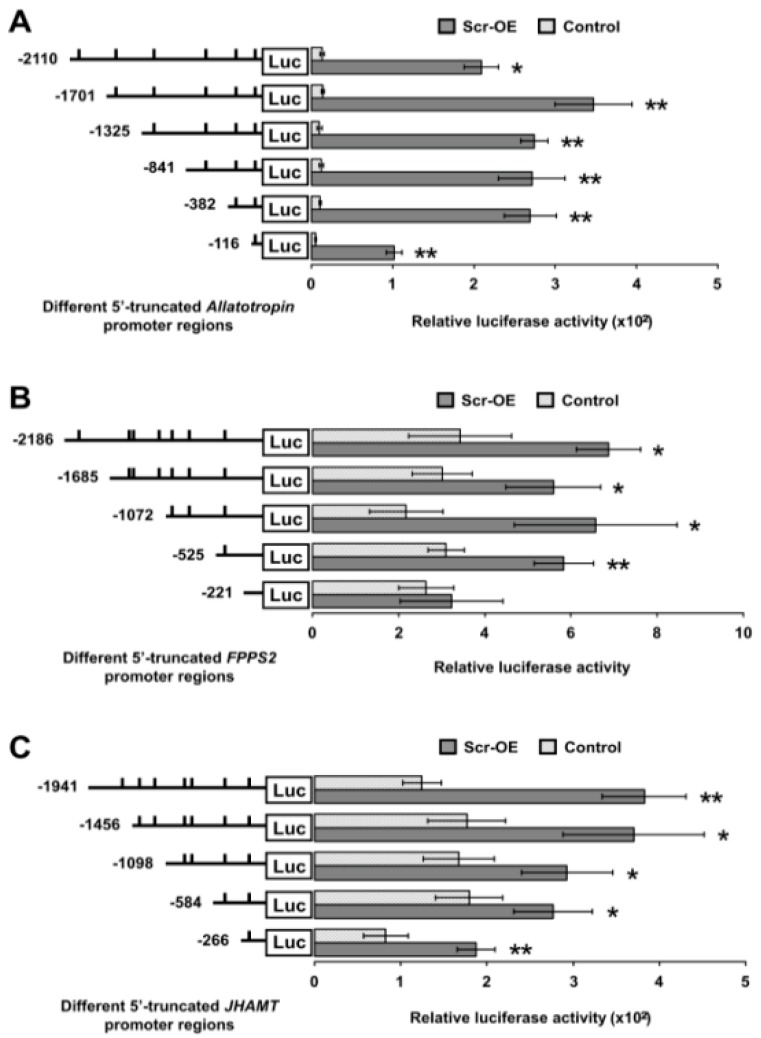
Truncation analysis of Scr-mediated activities of the promoters of genes involved in JH biosynthesis. (**A**) The effects of *Scr* overexpression on the luciferase expression driven by several 5′-truncated promoters of the *Allatotropin* gene. Luciferase reporter analysis in BmE cells indicated that every truncated promoter of *Allatotropin* significantly increased the luciferase expression after *Scr* overexpression; (**B**) The effects of *Scr* overexpression on the luciferase expression driven by several 5′-truncated promoters of the *FPPS2* gene. Luciferase reporter analysis in BmE cells indicated that the four longer truncated promoters of *FPPS2* significantly increased the luciferase expression after *Scr* overexpression, whereas the increase was not significant when the luciferase expression was driven by the shortest truncated promoter; (**C**) The effects of *Scr* overexpression on the luciferase expression driven by several 5′-truncated promoters of the *JHAMT* gene. The luciferase reporter analysis in BmE cells indicated that every truncated promoter of *JHAMT* significantly increased the luciferase expression after *Scr* overexpression. The predicted CREs for homeodomain transcription factors are indicated by short lines. Luc, luciferase; OE, overexpression. The error bars represent the mean ± S.E. (*n* = 3). The significance of the difference between data sets was calculated using two-tailed Student’s *t*-test. * *p* < 0.05; ** *p* < 0.01, compared with the control.

We further determined whether the Scr protein can directly bind to the promoter regions of genes involved in JH biosynthesis. The three JH biosynthesis-related genes (*Allatotropin*, *FPPS2*, and *JHAMT*) whose transcription was most significantly induced by *Scr* overexpression were selected for further analysis. To identify the authentic CREs for the Scr protein on the promoters of these JH biosynthesis-related genes, a series of luciferase reporter constructs driven by 5′ flanking truncated promoters upstream of the ATG start codon of each gene were generated. Further analysis in BmE cells indicated that every truncated promoter of both *Allatotropin* and *JHAMT* significantly increased the transactivation of luciferase expression following *Scr* overexpression, thereby suggesting that the potential CREs for the Scr protein in the promoter of these two genes might be located within the shortest promoter region (Figure 8A,C). In addition, for the *FPPS2* gene, the truncated promoter regions that included fragments that extended beyond position −525 significantly increased luciferase expression after *Scr* overexpression, but the truncations shorter than position −221 did not respond to *Scr* overexpression (Figure 8B), thereby indicating that the potential CREs for the Scr protein in the *FPPS2* promoter might be located in the region from position −525 to −221 (Figure 8B).

**Figure 9:**
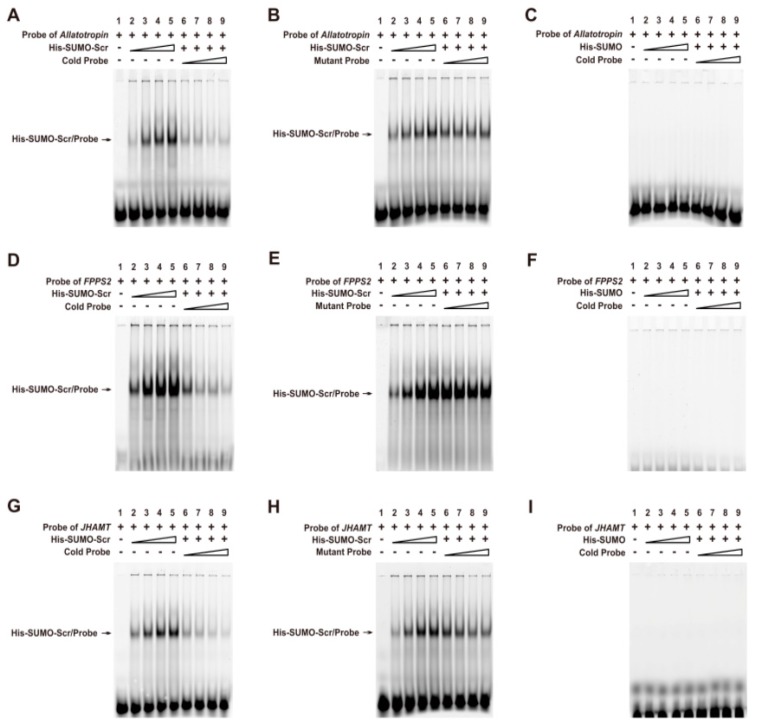
Direct binding of the Scr protein to the CREs in the promoters of genes involved in JH biosynthesis. (**A**,**D**,**G**) A series of EMSAs indicated that recombinant His-SUMO-Scr bound in a dose-dependent manner to the labeled probes in the promoters of *Allatotropin*, *FPPS2*, and *JHAMT*, and the binding could be competitively suppressed by the respective unlabeled probe; (**B**,**E**,**H**) The unlabeled mutant probes could not suppress the binding of the recombinant His-UMO-Scr to the labeled probes; (**C**,**F**,**I**) The His-SUMO tag alone could not bind to the labeled probes. The stepped shapes mean the increasing does of the recombinant proteins, the cold probes or the mutant probes respectively.

To confirm whether Scr can bind directly to the CREs on promoter regions of the three JH biosynthesis-related genes, we performed an electrophoretic mobility shift assay (EMSA) using the recombinant His-SUMO-Scr protein and 5′-Cy3-labeled oligonucleotide probes that contained the potential CRE for the Scr protein in the promoter of each gene (Table S3). As expected, the His-SUMO-Scr protein could bind directly to all three of the labeled probes in a dose-dependent manner, and this binding could be competitively suppressed by the unlabeled cold probes (Figure 9A,D,G). However, the unlabeled mutant probes could not competitively suppress this binding (Figure 9B,E,H). Moreover, the His-SUMO tag alone could not bind to the labeled probes (Figure 9C,F,I). These findings demonstrate that Scr regulates the transcription of genes involved in JH biosynthesis of the silkworm by directly binding to the CREs on their promoters.

## 3. Discussion

Silkworm moltinism varieties with different larval molt numbers are used as excellent models for studying the actions of either JH or ecdysone [5,6]. The dominant silkworm *M^3^* mutant undergoes three larval molts and exhibits a precocious metamorphosis [13,22]. Previous reports presumed that the JH titer may be higher in the *M^3^* mutant than in the WT strain which undergoes four larval molts at the feeding stage of the second instar [13,22]. Our data demonstrated that, compared with WT larvae, the *M^3^* mutant delayed the timing of the initiation of the second and the third moltings (Figure 1A). Importantly, the concentration of JH titer and the expression of the JH-responsive gene *Kr-h1* in JH-targeted tissue were greater during the second instar (Figure 2A,C), thereby indicating that the dominant *M^3^* mutant is caused by the enhanced JH biosynthesis activity and greater JH concentration in the second instar. This result differs from the JH deficiency in the recessive *mod* mutant with two larval molts and the ecdysone deficiency in the recessive *nm* mutant with no larval molting [6,12]. Moreover, the body weight of the *M^3^* larvae after the second molting increased and reached its final value more rapidly than did the WT larvae (Figure 1C). Given that a critical body weight during the last larval stage is an indicator for the onset of insect metamorphosis [14,15], we propose that the increased JH titer prolonged the duration of both feeding and growth in the second and third instars in the *M^3^* mutant, which in turn caused the larvae to rapidly reach a critical body weight that was appropriate for precocious metamorphosis, and this notion was strongly supported by our results of the induction of the trimolting silkworm by JHA application (Figure 3).

A striking finding of our study is that only the homeodomain transcription factor gene *Scr* is located in the genomic region that is linked to the *M^3^* locus, and it regulates the transcription of genes involved in the JH biosynthesis pathway. In the fruit fly, the Scr protein is required for not only the formation of both the posterior head and the anterior thorax but also for the development of the central and peripheral nervous systems [23,24,25,26]. In the silkworm, Scr has been shown to regulate the development of the anterior silk gland [27]. Our results strongly support the notion that Scr transcriptionally mediates JH biosynthesis in the silkworm. First, *Scr* expression was present in the Br-CC-CA complex, which is responsible for producing JH, and it exhibited a close correlation with the developmental changes in the JH titer in both the *M^3^* mutant and WT larvae (Figure 2 and Figure 5). Second, *in vitro* analysis demonstrated that Scr could regulate the transcription of genes involved in JH biosynthesis by directly binding to the CREs on their promoters (Figure 7, Figure 8 and Figure 9). To our knowledge, this is the first report of a homeodomain gene that is involved in JH biosynthesis in insects.

Although our results clearly indicate that Scr is transcriptionally involved in JH biosynthesis, the direct genetic evidence that Scr is responsible for the *M^3^* mutant is insufficient. To determine the genetic basis underlying the regulation of Scr in the *M^3^* mutant, we performed a knockout experiment for the *Scr* gene in the *M^3^* mutant using a transcriptional activator-like effecter nuclease (TALEN) approach and examined the change in the larval molt number because *M^3^* is a dominant strain, and JH production is higher in the *M^3^* than in the WT larvae. However, knockout of the *Scr* gene unexpectedly resulted in embryonic lethality. In addition, no obvious sequence variation occurred in the coding sequences of the *Scr* genes from *M^3^* and WT, and the variations in the 3′ UTR of the *Scr* gene were also not specific between the *M^3^* and WT strains (Figure S2). Intriguingly, obvious sequence variations could be identified within intron 2 of the *Sc*r gene between *M^3^* and WT (Figure 4C and Table S2) and these variations resulted from an insertion of the retrotransposon Bm1. Bm1 is a short interspersed nuclear element (SINE), which is a member of the most widespread and enriched class of eukaryotic transposable elements [28]. Previous studies have determined that adaptive insertions of transposable elements in the fruit fly genome are the major source of adaptive mutations [29]. A SINE insertion in intron 2 of the insulin-like growth factor 1 (*IGF1*) gene contributes to the domestication of dog breeds [30]. In the silkworm, an insertion of a retrotransposon that belongs to a long interspersed nuclear element (LINE) in the *cis*-regulatory region of the ecdysone oxidase gene increased its expression level [31]. Therefore, further investigation of the sequence variations within intron 2 of the *Sc*r gene between *M^3^* and WT strains may unravel the genetic basis for the roles of the *Scr* gene in the silkworm *M^3^* mutant.

## 4. Experimental Section

### 4.1. Silkworm Strains

The *M^3^* mutant strain *Shennong* undergoes three larval molts and forms four larval instars. The common WT strain *Dazao* undergoes four larval molts and forms five larval instars. These two silkworm strains are generally used in scientific research and are excluded from the endangered or protected species lists. The fertilized eggs were incubated at 25 °C with appropriate humidity for hatching, and the silkworm larvae were reared on fresh mulberry leaves at 25 °C under a photoperiod of 12 h of light and 12 h of dark.

### 4.2. Hormone Titer Determination

JH I (SciTech, Prague, Czech Republic), 20-hydroxyecdysone (Sigma-Aldrich, St. Louis, MO, USA) and trifluoroacetic acid (Nacalai Tesque, Kyoto, Japan) were dissolved in various solvents, including distilled water, acetone and hexane. Aluminum oxide (Sigma-Aldrich, St. Louis, MO, USA) was treated with 6% water to prepare activity grade III. Then [3H] ecdysone and ecdysteroid antiserum were stored in the lab of Professor Sheng Li (Shanghai Institutes for Biological Sciences, Chinese Academy of Science, Shanghai, China).

The extraction and derivatization of JH was performed according to the procedures described by Munyiri with slight modifications [32]. First, a sample of 100 μL of hemolymph was collected in a clean glass tube with 0.5 mL of methanol. Because the body size of the *M^3^* larvae in the second instar and that of the WT larvae in the second and third instars was too small to collect enough hemolymph, the samples of 0.1 g of whole bodies in these periods were respectively homogenized with 0.5 mL of methanol in a clean glass tube. Then, 1.5 mL of 2% NaCl and 0.5 mL of hexane were added. After mixing vigorously and centrifuging at 4000 rpm for 5 min, the hexane phase was collected. This extraction with hexane was repeated three times, and the combined solvent (1.5 mL) was evaporated under a stream of nitrogen. Then, 100 μL of methanol and 2 μL of trifluoroacetic acid were added to the crude extraction and incubated for 30 min at 60 °C to convert JH into its methoxyhydrine derivative (JH-MH). The treated sample was then loaded on an aluminum oxide column (activity grade III) that was prewashed with 1 mL of hexane, then washed with 2 mL of 30% ether in hexane and eluted with 2 mL of 50% ethyl acetate in hexane. Finally, the elution was concentrated to approximately 20 μL under a stream of nitrogen and subjected to gas chromatography-mass spectrometry (GC-MS) analysis.

The JH I level was measured using 7890B/5977 GC-MS system (Agilent, Santa Clara, CA, USA) equipped with an HP-5 MS column (0.25 mm × 30 m). The injector and detector temperatures were maintained at 300 °C and 330 °C, respectively. The column temperatures were initially held constant at 120 °C for 2 min and increased first to 200 °C at 20 °C/min and then to 320 °C at 15 °C/min. Derivatized JH I was monitored at *m*/*z* = 90.1 and appeared after 6.8 min of retention.

Ecdysone was extracted and measured by radioimmunoassay (RIA) examination according to previously described methods [33]. Briefly, 0.5 mL of hemolymph or 0.5 g of whole bodies was collected or homogenized with 0.5 mL of methanol in a glass tube, and then mixed vigorously. After centrifuging, the methanol phase was transferred to a new tube. The extraction was repeated, and the combined methanol phase (1 mL) was incubated for 30 min on ice and then centrifuged. The supernatant was further evaporated in a dry bath at 70 °C and the remainder was dissolved in 50 μL of borate buffer (0.05 M H_3_BO_3_, 0.9% NaCl, and 0.05% Triton X-100, pH = 8.4). Meanwhile, [^3^H] ecdysone (3000 DPM/sample) and ecdysteroid antiserum (1:16,000 dilution, 10 μL/70 samples) with 150 μL of borate buffer were incubated at 28 °C for 30 min. Then, a 50× diluted sample was added, and the admixture (200 μL) was incubated at 28 °C for at least 2 h until the competing binding to ecdysteroid antiserum by 20E and [^3^H] ecdysone in the sample was balanced. Next, the mixtures were further incubated at 4 °C overnight. To isolate the amount of radioligand that was bound to the antiserum, 50 μL of dextran-coated charcoal (DCC) (1 g of charcoal in 100 mL of dextran buffer), which was capable of absorbing the free radioligand, was added, and the mixtures were incubated on ice for 5 min. The supernatant was obtained by centrifugation at 2000× *g* for 5 min, and 400 μL of supernatant was transferred to a new, clean glass tube with 2 mL of scintillation fluid and incubated at room temperature for 1 h. The amount of bound radioligand was quantified using a liquid scintillation counter (Beckman Coulter, Indianapolis, IN, USA).

### 4.3. JHA Application

The juvenile hormone analogue (JHA) methoprene (Sigma-Aldrich, St. Louis, MO, USA) was diluted with 50% acetone to a concentration of 1 mg/mL, and 1 μL of the methoprene per larva was topically applied to newly molted second instar larvae along the dorsal midline of the larval thorax as previously described [34]. The larvae treated with 50% actone at the same time point were used as control.

### 4.4. RNA Extraction and qRT-PCR Analysis

Total RNA from the brain-corpora cardiaca-corpora allata (Br-CC-CA) complex and the prothoracic gland (PG) from different larval stages was isolated using the PureLink RNA Micro Kit (Invitrogen, Carlsbad, CA, USA) according to the manufacturer’s protocol. Total RNA from the silkworm embryo-derived (BmE) cells was isolated using the Total RNA Kit II (Omega Bio-Tek, Norcross, GA, USA). Then, 1 μg of total RNA were reverse transcribed using an oligo(dT)_18_ primer and Moloney murine leukemia virus reverse transcriptase (Promega, Madison, WI, USA).

Quantitative RT-PCR (qRT-PCR) analyses were performed using the 7500 Fast Real Time PCR System (Applied Biosystems, Foster City, CA, USA) with the SYBR Premix EX Tap Kit (Takara, Otsu, Japan) according to the manufacturer’s instructions. The relative mRNA levels of the target genes were calculated using the 2^−ΔΔ*C*t^ method [35] and the qRT-PCR protocol was as follows: denaturation at 95 °C for 3 min followed by 40 cycles of 95 °C for 15 s, 60 °C for 30 s, and 72 °C for 30 s. The silkworm eukaryotic translation initiation factor 4A (*eIF-4a*) gene was used as the internal control. The primers used for qRT-PCR are listed in Table S1.

### 4.5. Genomic DNA Extraction

Genomic DNA of the parental strains and F1 individuals was isolated from adult heads, and genomic DNA of backcross 1 (BC1) individuals, which were obtained by crossing WT females with F1 heterozygote males obtained from crossing of the *M^3^* mutant and WT, was isolated from the heads of larvae using DNAzol (Invitrogen, Carlsbad, CA, USA) according to the manufacturer’s protocol. The genomic DNA samples were purified with PI-1100 (Kurabo Industries, Osaka, Japan).

### 4.6. Positional Cloning

To construct the *M^3^* linkage map, single nucleotide polymorphism (SNP) markers were identified at various positions on chromosome 6. The markers that presented polymorphisms between *M^3^* and WT strains were selected to examine the genotype of BC1 individuals with *M^3^* moltinism phenotypes. The PCR protocol was as follows: initial denaturation was performed at 95 °C for 5 min. This was followed by 40 cycles of varying the temperature in the following manner: 95 °C for 10 s, 55 °C for 15 s, and 72 °C for 30 s. Subsequently, a temperature of 72 °C was maintained for 7 min. The primers for the SNP markers that were used in the linkage analysis are listed in Table S1. Based on the results of SNP mapping, the candidate gene that was responsible for the *M^3^* locus was annotated using the SilkDB (http://silkworm.swu.edu.cn/silkdb/), KAIKObase (http://sgp.dna.affrc.go.jp/KAIKObase/) and National Center for Biotechnology Information (NCBI; http://www. ncbi.nlm.nih.gov) databases.

### 4.7. Plasmid Construction

The vector pSLfa1180fa was modified by inserting the *hr3* enhancer and the proximal promoter region of the silkworm *Act4* gene on the base of the transgenic vector created by Horn and Wimmer [36]. The open reading frame (ORF) of silkworm *Scr* was amplified with an N-terminal *Flag*-tag using the primer (*Flag*-*Scr*) listed in Table S1, and the sequence was subsequently cloned into the pSLfa1180fa vector to construct the *Scr* overexpression vector (1180-*hr3*-*A4*/*Flag*-*Sc*r) for overexpressing *Scr* in BmE cells. The ORF of enhanced green fluorescent protein (*EGFP*) gene was also cloned into the pSLfa1180fa vector to construct the *EGFP* overexpression vector (1180-*hr3*-*A4*/*EGFP*) for overexpressing *EGFP* in BmE cells as the negative control.

The promoter-specific primers (*Allatotropin*-pro-2110, *HMGS*-pro, *HMGR*-pro, *MevK*-pro, *MevPPD-*pro, *FPPS2*-pro-2186, *JHAMT*-pro-1941) listed in Table S1 were designed for cloning the potential promoter sequences of the JH biosynthesis-related genes, including *Allatotropin*, *HMGS*, *HMGR*, *Mevk*, *Mevppd*, *FPPS2* and *JHAMT*. The 5′-truncated promoter sequences of *Allatotropin*, *FPPS2* and *JHAMT* were also cloned using the primers (*Allatotropin*-pro-1701, *Allatotropin*-pro-1325, *Allatotropin*-pro-962, *Allatotropin*-pro-382, *Allatotropin*-pro-114 for *Allatotropin*; *JHAMT*-pro-1466, *JHAMT*-pro-1098, *JHAMT*-pro-584, *JHAMT*-pro-266 for *JHAMT*; *FPPS2*-pro-1685, *FPPS2*-pro-1073, *FPPS2*-pro-525, *FPPS2*-pro-221 for *FPPS2*) listed in Table S1. After restriction enzyme digestion and purification, the fragments were cloned into the plasmid pGL3-basic (Promega, Madison, WI, USA) and used to construct the luciferase reporter vectors.

### 4.8. Cell Culture and Transfection

BmE cells were cultured in Grace’s medium (Thermo, Waltham, MA, USA) supplemented with 10% HyClone fetal bovine serum (GE Healthcare Life Sciences, Logan, WV, USA) at 27 °C [37]. Transfection of the recombinant vectors was performed using the Cellfectin II reagent (Thermo, Waltham, MA, USA). Quantitative RT-PCR analysis for the target genes was performed as described above, and the luciferase activities were measured using commercially available kits (Promega, Madison, WI, USA) according to the manufacturer’s instructions.

### 4.9. Western Blotting

To test whether the Scr protein was successfully overexpressed in BmE cells, we performed Western blotting analysis. The BmE cells were extracted in lysis buffer (1% Triton X-100 in PBS, pH = 8.0), and the protein concentrations were estimated using a bicinchoninic acid (BCA) assay (Beyotime, Shanghai, China). For Western blotting, 10 μg of protein per sample was separated using 12% sodium dodecyl sulfate polyacrylamide gel electrophoresis (SDS-PAGE) and subsequently transferred to a polyvinylidene difluoride (PVDF) membrane (GE Healthcare Life Sciences, Logan, WV, USA). The membranes were blocked in 5% bovine serum albumin (BSA) diluted in TBST (10 mM Tris-HCl, 150 mM NaCl, 0.05% Tween20, pH = 7.5) at 4 °C overnight and incubated with primary antibodies targeting Flag or α-tubulin (Sigma-Aldrich, St. Louis, MO, USA) at a 1:15,000 dilution for 1 h at 37 °C. After rigorous washing, the membranes were then immunoblotted with an anti-mouse IgG (H+L)-peroxidase antibody diluted 1:20,000 (Sigma-Aldrich, St. Louis, MO, USA) for 1 h at 37 °C. The signal was visualized via the chemiluminescence method using the SuperSignal West Femto Maximum Sensitivity Substrate (Thermo, Waltham, MA, USA).

### 4.10. Recombinant Expression and Purification

The ORF of silkworm *Scr* gene was amplified using the primers listed in Table S1 and then cloned into the prokaryotic expression vector pCold-SUMO (Haigene, Harbin, China). The recombinant vector pCold-SUMO/Scr was transformed in *E. coli* strain *BL21* (*DE3*) competent cells (TransGen, Beijing, China). The *E. coli* cells were then induced by 0.2 mM isopropyl b-d-1-thiogalactopyranoside (IPTG) at 16 °C for 20 h in a Luria-Bertani medium that contained 20 μg/mL ampicillin to express the recombinant Scr protein, which was subsequently purified using Ni-NTA affinity columns (GE Healthcare Life Sciences, Logan, WV, USA), according to the manufacturer’s instructions, to be used in binding assays.

### 4.11. EMSA

To test whether Scr can bind to the *cis*-regulatory elements (CREs) of homeodomain transcription factors on the promoters of the genes involved in JH biosynthesis, electrophoretic mobility shift assays (EMSAs) were performed as previously described [38,39]. The DNA oligonucleotides (listed in Table S3) that contained the consensus binding sites (represented as red letters in Table S3) of the homeodomain transcription factors were labeled using Cy3 at the 5′-end and annealed to produce double-stranded probes. The DNA-binding reactions were performed using 0.1–1 μg of the purified recombinant Scr with 3 μL of 5× binding buffer (Beyotime, Shanghai, China) in a volume of 15 μL. The labeled probe (5 μM) was added after incubation for 20 min at 25 °C, and the incubation was continued for an additional 20 min. For competition assays, a 5–50× unlabeled or mutant double-stranded probe was added to the reaction before the labeled probe was added. After the addition of 2 μL of 10× loading buffer (Beyotime, Shanghai, China), the mixtures were subsequently loaded onto 5% polyacrylamide gels and electrophoresed in 1× TBE buffer (45 mM Tris-borate and 1 mM EDTA, pH 8.3). Finally, the gels were scanned and imaged using an Amersham Typhoon scanner (Thermo, Waltham, MA, USA).

### 4.12. Statistical Analysis

Error bars represent the mean ± S.E. and the significance of the difference between data sets was calculated using two-tailed Student’s *t*-test in this study.

## 5. Conclusions

The silkworm *dominant trimolting* (*M^3^*) mutant undergoes three larval molts and exhibits precocious metamorphosis. In this study, we observed that, compared with the WT larvae, the concentration of JH titer is greater during the second larval instar of the *M^3^* mutant. Positional cloning analysis indicated that only the homeodomain transcription factor gene *Scr* is located in the genomic region that is tightly linked to the *M^3^* locus. Furthermore, luciferase reporter analysis and EMSA examinations demonstrated that the Scr protein regulates the transcription of genes involved in JH biosynthesis by directly binding to the CREs on their promoters. This is the first report regarding the role of the homeodomain factor in insect JH biosynthesis and establishes a solid foundation for further unraveling the genetic basis of the silkworm *M^3^* mutation.

## Supplementary Materials

Supplementary materials can be found at http://www.mdpi.com/1422-0067/16/11/25945/s1.
