# Supplementary material for: Homeodomain Protein Scr Regulates the Transcription of Genes Involved in Juvenile Hormone Biosynthesis in the Silkworm
Source: Int J Mol Sci. 2015 Nov 2;16(11):26166–85. doi: 10.3390/ijms161125945 (PMC4661804; doi:10.3390/ijms161125945)
Supplement: Supplementary file 1 [file ijms-16-25945-s001.pdf]

International Journal of *Molecular Sciences*

Supplementary Information

Table S1. The primers used in this study.

Purpose	Primer Name	Sense Sequence (5'–3')	Anti-Sense Sequence (5'–3')
Linkage analysis	S002	CGCTGGTAGCCTAAGTTGC	GGACAGAGCACCATAACAGTAG
	S011	TTTGGTTACAGATTTCTTGATTG	TCTATTCAACGGGAGTGGG
	S012	CCAGTGCGGCAAAAGTTAC	GCGGAGCATTACTACAGAACAT
	S013	GAGTAAAAGAGCCCGTTCAA	CATCCTTCAAACGAGAACACT
	S016	AGGAAGCCAAGTCGTTTATG	AACATTCTAATCCGAGTCCTG
	S020	CGATCTTGGTGAAACCTGTG	TGCTTTTGAGCCATCTACG
	S031	TCATTTTATGTCTACACGCTTCC	CACCCGACTTGCTCACATAC
	S048	ACTGCTCAAGGCAGAAATGG	TCACTCTGAAGAGGTTCAAGAAG
	S105	AATAAAACATCACTGCCCCG	TCCGACAGCAAACTTAGCGT
	S113	AAGCGGACTACATACCACCAG	GCTCCAGCGACATGTATCTTC
	S117	AGAATGAAATGGAAGAAGGATA	CGACACAAAAGGAATGGTAAG
	S119	GTGATGGCAAACACAACTACA	TTTGACTTGTCCACAGAGCAG
	S126	CAAAGGGTGGATGTTATGCT	GTGATGTGTAAATGGGGCAA
	S147	ATTCAATCAGAAACGCCCTCA	GCTCCTAAACAGTCGCCAGA
	S186	GCTGTTGAGACTGCCTTTG	GCTGATACGATTCCGATGA
	S192	AATCAAAATAGGTGAAGGTGG	TATGGAAAGTAGCAGTCAGGA
	S194	CAAATATCGATGAATGCACAA	GGTGAGTTTATTGCCGATTTA
Sequence cloning	*Scr*-ORF	ATGAACGACTTAAATTACAACGC	CTATGCGGAGAGATGGGC
	Scr-3′UTR-1	AGTATCGAATATGCATTTTGAGA	TCATACGAGATTTGTGTAGTGTT
	Scr-3′UTR-2	ACAAAATGAGAGTGAAACAAGAC	CCATACCAATTCGACCTTCAC
	Intron2-01	GACATTAGATTATTACCTTCTTC	GCTCCTAAACAGTCGCCAGA
	Intron2-02	AAGTCCATTCGCATTTTTCAT	CATCCGAGACGAGTTGTGTAT
	Intron2-03	ATTCCACGGGCTGGCTCTT	CATCAGTATTTTCTTATGACGCT
	Intron2-04	CGTGTTGCATGGTCGAATTTT	GCACCGTCTGCCTGTAATGG
	Intron2-05	CGGACGTAAAAACAGCGATAT	CGGACACAGTTTGACAGTTTTA
	Intron2-06	TGTAAATAATAGCACAACGCAA	ATAAATCAAACAATCGCACG

Table S1. *Cont.*

Purpose	Primer Name	Sense Sequence (5'–3')	Anti-Sense Sequence (5'–3')
	Intron2-07	GAGAGCTCGGACTTTCCTGTC	GTTATGCCGCACTAAGAAAAT
	Intron2-08	TCGCATACAGGAGAGTACTAATACC	ACGTTCTACGCAGAACGCAGG
	Intron2-09	CTGGCTTTTAATCTTAGTGGG	CGAATACGAAACTACCCGACA
	Intron2-10	TAGCATACAGGAGTGAACGAG	AGTTACCAAGAAGGGCTACG
	Intron2-11	CTGCGAGAAACTTGATGGGT	GATGCATTACCCTGGGAACTC
	Intron2-12	TCCTTTGATCGTGTTTGGATG	CAACCCTGCAATGTTCTAATC
	Intron2-13	AATTCGGTGAAAATCCTGAAA	CCGAATATCATTTGCACCTA
	Intron2-14	GACGCTAAAGCCAGACTAAC	ACGGCCGCTATAAAAGTCTAA
	Intron2-15	CAGAGTCGTTAGAACATCGCC	GAACATACGATGCATTACCA
	Intron2-16	CGAAGCCGAACTTACTATCT	AATTACCTAATCATCGCGAGC
	Intron2-17	AAGTAAGTTTTGGTGACGGAC	GGTGATATTTTGCTTTTCGCT
	Intron2-18	GAAAAGGCTCTGACAGGACATA	GCTGCCAACTGAAAAATACTAT
	Intron2-19	GTATCGTGGCTTTGTGGCTTC	AAAACTTGGTGGGTCTTAGCC
Sequence cloning	Intron2-20	TTGTGCCAATGGATTATGAT	TAGAACCGGTGATGAAGCTTA
	Intron2-21	TATACGCACCGTTCGACTCT	AGTTCTTATTCGGTCGACATT
	Intron2-22	AACTACATTGTTTGGCTTTCC	AAGCGAGCCCAATCTGTCTAC
	Intron2-23	TGAGTTATGAAGGCGGTCGG	TCAATCGGATGCTTATCAAAAA
	Intron2-24	ACCAGCCCTGATAATATGTATCA	TCGATCTCTAAAACGATTGCT
	Intron2-25	ATTAGGTGTTTGGCTCTGAGA	CTGTGAGGCAGTGGAAAAACC
	Intron2-26	GGTATTGTCCATGAGGTAACG	GGACCACCTATCCTGCTCTG
	Intron2-27	CCATCGCCATCTTGCCTTA	TCCAGGCTCTATCCTTCTTAT
	Intron2-28	GGCTTACGCCATAACTACATA	TTAAATTGGTAAGCAGCGACT
	Intron2-29	TCTTCAGAGTAACAAAACCGC	GCTCAAAGGGTTAAGGGTTCT
	Intron2-30	CTCTTCGAACTTGTCAACTCC	GTTGCCATGCGTTCGTTAAGA
	Intron2-31	AATGCTACATGACGACCGTGA	CTTTCTCGCTGTACAAAGATG
	Intron2-32	GCAGAGTTTTTGGTGACCTTTG	TGCGCGACGATAACCTATAATT
	Intron2-33	CACATCACTAAAGAAGCCCGC	GATTAAATCGAGCCGCCAACA

Table S1. *Cont.*

Purpose	Primer Name	Sense Sequence (5'–3')	Anti-Sense Sequence (5'–3')
Sequence cloning	Intron2-34	GCTCAGTGGGCTAAGCATCAG	GACTATCCAAAGCGTGGTTCT
	Intron2-35	TTTATGACTAGCTTTCGCCCG	AACTGTGAATACCGCAGACCC
	Intron2-36	GATTGTCATCCCAAATTCTACCA	TAATTTGCGTAAACTGGGCTA
	Intron2-37	GCAGCAAAAACTAGAGATGGAA	TGCTGTGAGAAATGCCACTATT
	Intron2-38	CGAAGCCTCAGAAAGAATATGG	CCGTGAAATCATAAACGACTTG
	Intron2-39	TTTATCTGTTACATCAAACCGTG	AACGACTTAAATTACAACGCC
qRT-PCR	*Scr*	CAAGTCTTCGGCTAACTCGCA	GTCCTCTGACGTTTCGTCTCG
	Kr-h1	ACCCATACTGGCGAGCGACCAT	CCTCTCCTTTGTGTGAATACGACGG
	EcR	GCTGGTCTGATAACGGTGGCT	CAAGGATTCCGGCGACATAAC
	Allatotropin	GAGATGATGACCGCCAGGG	GAACCAGTCCAGAGGGATGCT
	HMGS	TTGTTTTCATACGGTTCAGGATTGG	AACTTACTGGGTTCCACACTCTGCC
	HMGR	GAAGCGGAGTATCAAGCAGCC	CCACCAACAGAAAGAGAAACGG
	MevK	ACGAACCAGCAGTCCACATACA	GGCAACGAGTCAAAGTTAGGCT
	MevPPD	CAACGTCACCAGAAAACTTCATAACAGGTT	CCCGTTACTGTCTAGCAAATGTGAATCTA
	FPPS2	CATAGACTGCTTCGGTGATGAAATAAAA	TTCACTGCTACCATAACAGGCTTTGA
	JHAMT	TGGCTGCGACATAAGCGAAGA	CCTTGTTTCAGGTCTGCGGTCAA
	PTTH	AAACTCTGTTCCACGCTTCATTG	TCCTGCGATTTAGTTTCCCTTC
	PTSP	AGATGACAAGAGAGCCTGGAGC	CTCCATAGCCTCATCATCATCG
	Phantom	AAAAACGAATCGCTTCAGGAGTA	TGTATTTGACGAAACCATTGCC
	Shadow	TCGAGGAAGGGACTCCAGTAATAGC	CAAATGGCAGTGTGGCAGATGGTAC
	eIF-4a	TTCGTACTGCTCTTCTCGT	CAAAGTTGATAGCAATTCCCT

Table S1. *Cont.*

Purpose	Primer Name	Sense Sequence (5'–3')	Anti-Sense Sequence (5'–3')
Plasmid construction	*Flag-Scr*	CGCGGATCCATGGATTATAAAGATGATGATGATAAAAACGACTTAAATTACAA	AAATATGCGGCCGCCTATGCGGAGAGATGGGC
	EGFP-Scr	CGCGGATCCATGAACGACTTAAATTACAACGC	ATAAGAATGCGGCCGCCTATGCGGAGAGATGGGC
	Scr-pCold-SUMO	CGCGGATCCATGAACGACTTAAATTACAACGC	CCCAAGCTTCTATGCGGAGAGATGGGC
	Allatotropin-pro-2110	TCCCCCGGGCTTAACATCAGGTGGGCTGTGA	CCCAAGCTTGTGGTCTCGCAACTTTTCCG
	Allatotropin-pro-1701	TCCCCCGGGGTCTAAAGCAAGCGGTGCCTAA	CCCAAGCTTGTGGTCTCGCAACTTTTCCG
	Allatotropin-pro-1325	TCCCCCGGGTGGACCGAAAATGTCAATGC	CCCAAGCTTGTGGTCTCGCAACTTTTCCG
	Allatotropin-pro-962	TCCCCCGGGGCTTTTTGATTATGACCGAT	CCCAAGCTTGTGGTCTCGCAACTTTTCCG
	Allatotropin-pro-382	TCCCCCGGGATGGCTCTTGTAACATTTTCTG	CCCAAGCTTGTGGTCTCGCAACTTTTCCG
	Allatotropin-pro-114	TCCCCCGGGAATTAGCGTAGCACGCTGTA	CCCAAGCTTGTGGTCTCGCAACTTTTCCG
	HMGS-pro	TCCCCCGGGGAAGGGTAAAGTGGTTTGAAGAGT	CCCAAGCTTTATACTGCTTTCACAATATGAATTG
	HMGR-pro	TCCCCCGGGTTAGGTACCACAGCATCCGGTAA	CCCAAGCTTTTTCTGATATCTGAAACGAAAGAGG
	MevK-pro	TCCCCCGGGACACGCACACACTCTCTCTCTC	CCCAAGCTTTGTCCTTTTAATGTAATAAGCACAC
	MevPPD-pro	TCCCCCGGGCCACTACGCCCTAGAGGTCTT	CCCAAGCTTTTTTAAGAAAAAAAGGAATGGCT
	FPPS2-pro-2186	TCCCCCGGGGCTTACAATACGCCTTACAACCA	CCCAAGCTTGTCTACTATGTCATGAAGTCCAATG
	FPPS2-pro-1685	TCCCCCGGGTGTACAAATTCAGGGCTGTCATC	CCCAAGCTTGTCTACTATGTCATGAAGTCCAATG
	FPPS2-pro-1073	TCCCCCGGGATAAAACTCACTGTTCCAAAATCG	CCCAAGCTTGTCTACTATGTCATGAAGTCCAATG
	FPPS2-pro-525	TCCCCCGGGATAACCTTCGCAAGTGAAAACA	CCCAAGCTTGTCTACTATGTCATGAAGTCCAATG
	FPPS2-pro-221	TCCCCCGGGCCTCATTTTGGTGTATTCCTTT	CCCAAGCTTGTCTACTATGTCATGAAGTCCAATG
	JHAMT-pro-1941	ACCGAGCTCAATACCGCCACCCACCTTG	TCCCCCGGGACAGTCTTTGCAGGGAGCG
	JHAMT-pro-1466	ACCGAGCTCATGGTTTCCGCTGAGTTTT	TCCCCCGGGACAGTCTTTGCAGGGAGCG
	JHAMT-pro-1098	ACCGAGCTCGATTGGTAGTGCCTCCGAAGAC	TCCCCCGGGACAGTCTTTGCAGGGAGCG
	JHAMT-pro-584	ACCGAGCTCTCGGTGACGTCATACGTTCAG	TCCCCCGGGACAGTCTTTGCAGGGAGCG
	JHAMT-pro-266	ACCGAGCTCTTTTACCGAATTTCAAAACTGT	TCCCCCGGGACAGTCTTTGCAGGGAGCG

Table S2. Sequence variations in intron 2 of *Scr* between M^3 mutant and WT.

Region	Location in WT	Length in WT (bp)	Length in M^3 (bp)	Mutation in M^3	
				Insertion (bp)	Deletion (bp)
Region 1	2573614 ~ 2575475	1862	2181	319	—
Region 2	2580531 ~ 2583278	2748	2880	132	—
Region 3	2595166 ~ 2596976	1811	1499	—	312
Region 4	2597347 ~ 2600417	3071	2011	—	1060
Region 5	2600640 ~ 2603260	2621	2900	279	—
Region 6	2618304 ~ 2620972	2669	2443	—	226

Table S3. Oligonucleotide probes used in EMSA**.**

Gene	Probe	Sequence	Length (bp)	Location
Allatotropin	Labeled	5'-CCATAGGTTTGTAGATAATTAGCGTAGCACGCTGTA-3'	36	−94–−129
	Mutant	5'-CCATAGGTTTGTAGACCCCCAGCGTAGCACGCTGTA-3'		
FPPS2	Labeled	5'-TCGCAAGTGAAAACAATTAATTCGCAATCGGTTCA-3'	35	−482–−516
	Mutant	5'-TCGCAAGTGAAAACACCCCCTTCGCAATCGGTTCA-3'		
JHAMT	Labeled	5'-ATGGCTAATTTTTTTAATGAATTAAAAGAAC-3'	31	−124–−154
	Mutant	5'-ATGGCTAATTTTTTCCCCGAATTAAAAGAAC-3'		

Red letters represent the core-binding sites; gray letters represent the mutated binding sites.

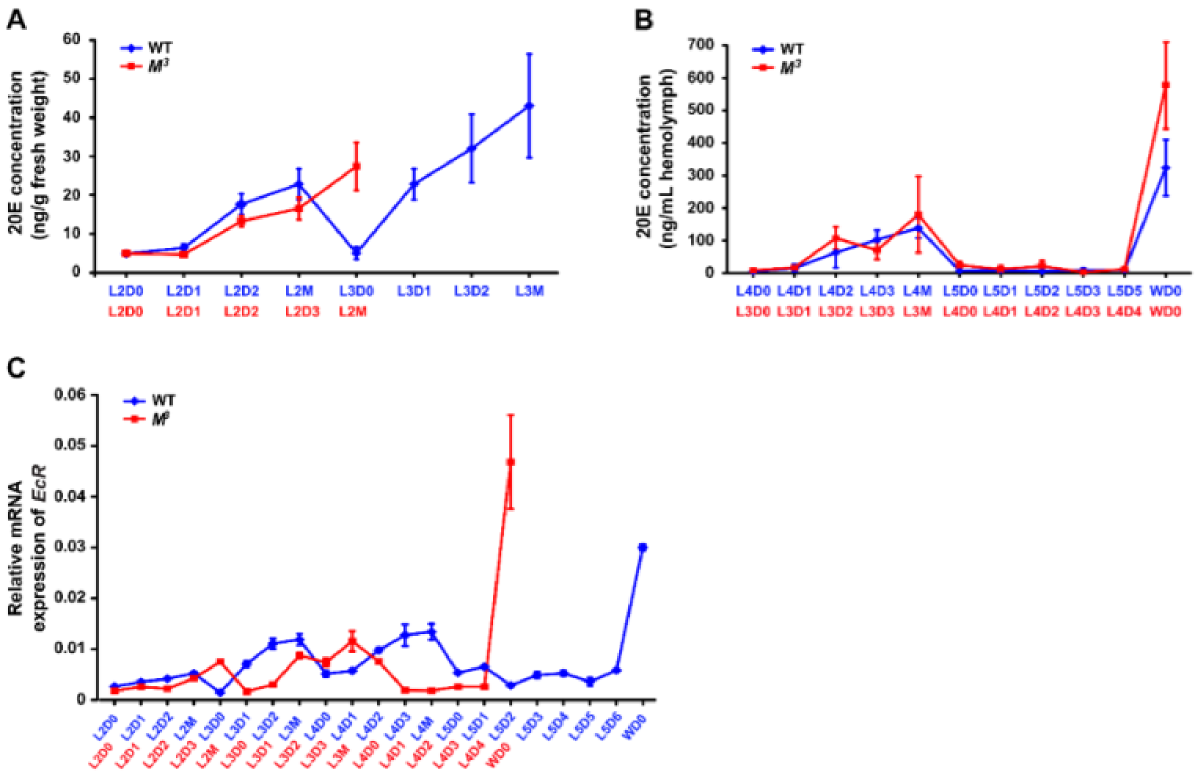

Figure S1. Ecdysone concentration and developmental expression of the *EcR* gene in the *M*^{3} and WT larvae. (**A**) Ecdysone concentration in the whole bodies of the *M*^{3} and WT larvae during early instars. RIA analysis indicated that the ecdysone titers in the whole bodies were similar between the *M*^{3} and WT larvae in the second instar; (**B**) Ecdysone concentration in the hemolymph of the *M*^{3} and WT larvae during late instars. RIA analysis indicated that during late instars, a major peak of the ecdysone titer in the hemolymph was present before the final larval molts and at the beginning of wandering in both the *M*^{3} and WT larvae; (**C**) *EcR* expression in the integument of the *M*^{3} and WT larvae. qRT-PCR analysis confirmed that the expression of the *EcR* gene at the beginning of the second larval molting in the WT larvae was similar to that at the same time point in the *M*^{3} larvae and exhibited a close correlation with ecdysone titer changes. L, larval instar; L2 to L5 respectively represents the second to the fifth larval instar; D, day; D0, initial day; D1 to D6 respectively represents the first to the sixth day of a larval instar; M, molting; W, wandering. The error bars represent the mean ± S.E. (n = 3).

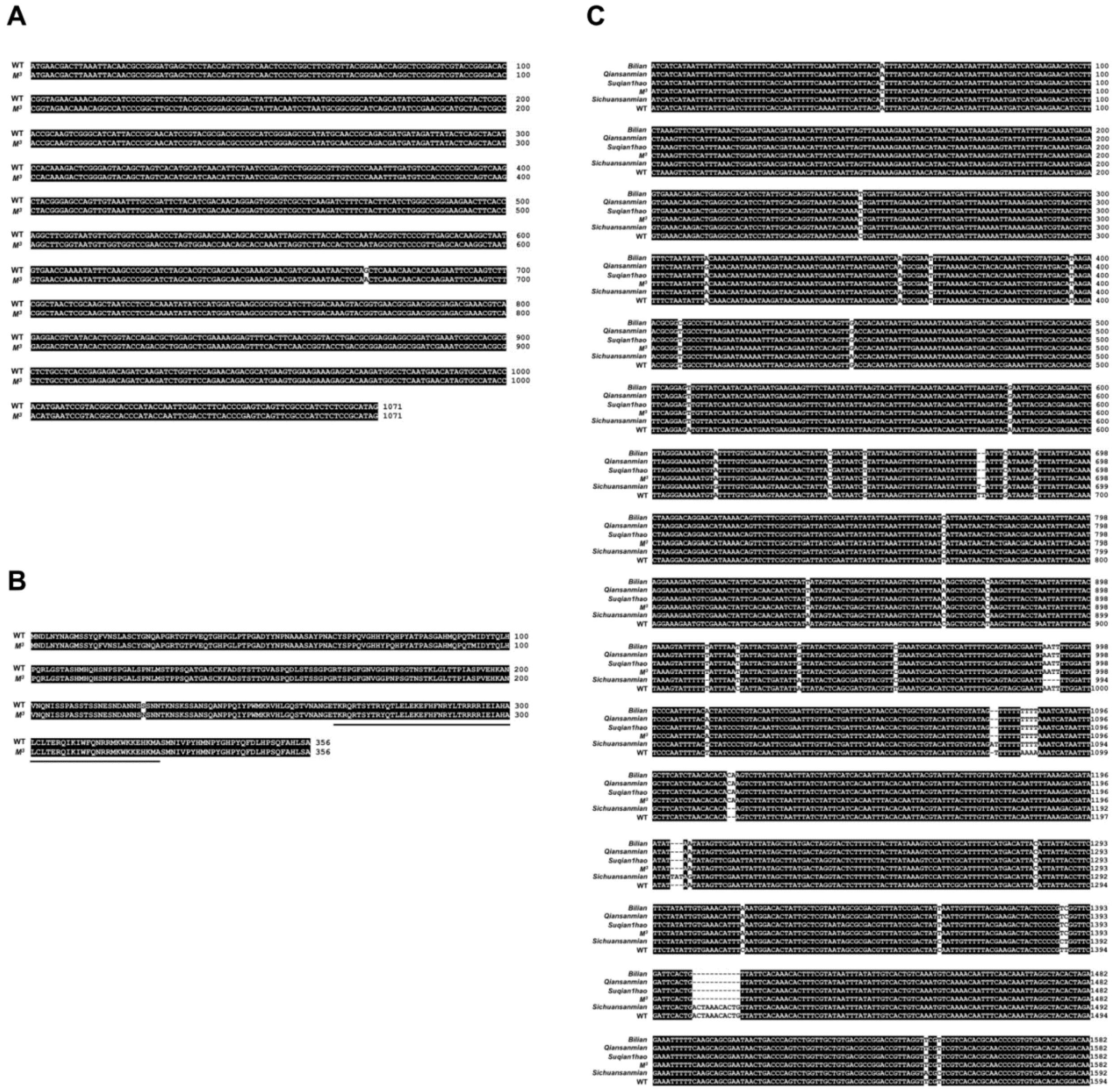

Figure S2. Sequence comparison of the *Scr* gene between *M*^{3} and WT strains; (**A**) Only one nucleotide in the coding sequence of *Scr* differed between the *M*^{3} and WT strains; (**B**) Only one amino acid of Scr was altered in the *M*^{3} mutant. The black lines indicate the region of homeodomain; (**C**) The alignment results revealed that the differences in the 3′UTR of *Scr* between *M*^{3} and WT strains were also present in other silkworm strains with three or four larval molts. The silkworms *Qiansanmian*, *Suqian1hao*, and *Sichuansanmian* are trimolting strains that are similar to the *M*^{3} mutant; *Bilian* is a tetramolting strain that is similar to the WT strain.

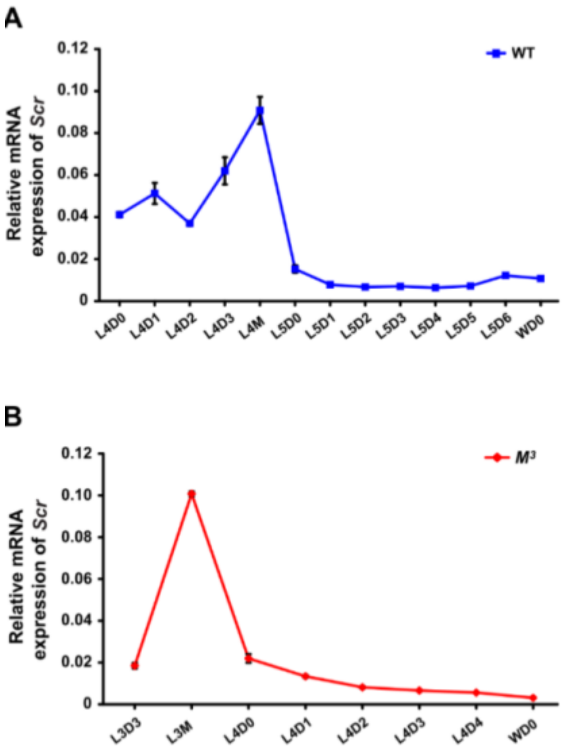

Figure S3. Developmental expression of *Scr* in the PG of the *M*³ and WT larvae during their late instars. qRT-PCR analysis indicated that the *Scr* expression was high during the final larval molting but very low before the larval-pupal metamorphosis in both the WT (**A**) and *M*³ (**B**) larvae. Furthermore, *Scr* expression did not correlate with ecdysone biosynthesis. L, larval instar; L3 to L5 respectively represents the third to the fifth larval instar; D, day; D0, initial day; D1 to D6 respectively represents the first to the sixth day of a larval instar; M, molting; W, wandering. The error bars represent the mean ± S.E. (n = 3).

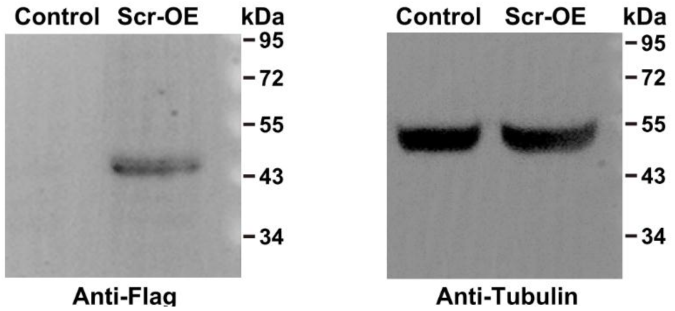

Figure S4. Western blotting analysis indicated that the Scr protein was successfully overexpressed in BmE cells. OE, overexpression.

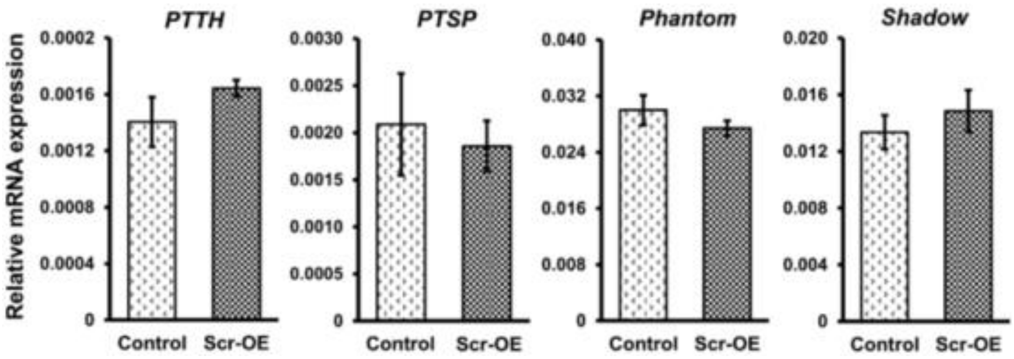

Figure S5. *Scr* overexpression could not induce the expression of genes involved in the ecdysone biosynthesis pathway. Expression profiles of genes involved in ecdysone biosynthesis, including *PTTH*, *PTSP*, *Phantom*, and *Shadow*, could not be induced by *Scr* overexpression. OE, overexpression. The error bars represent the mean ± S.E. ($n = 3$).

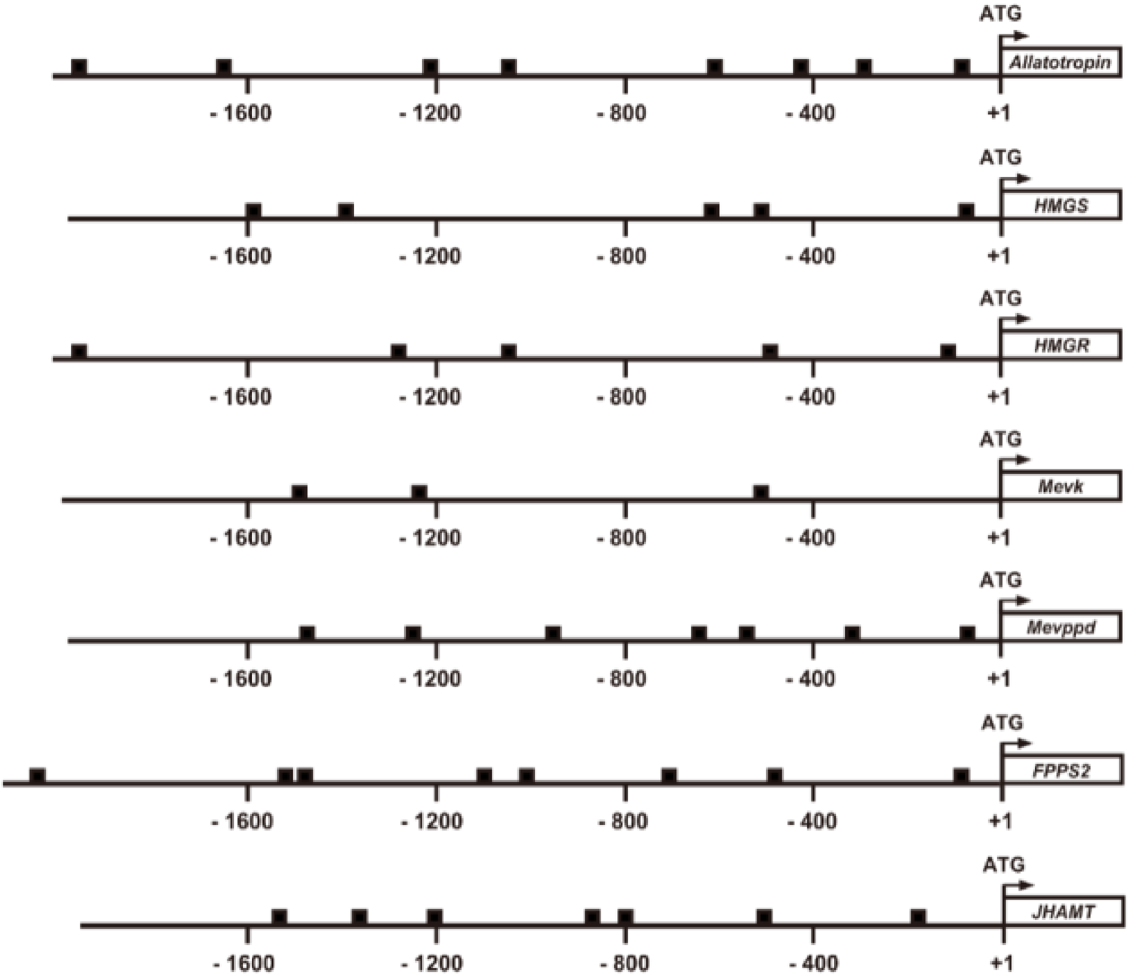

Figure S6. CREs for homeodomain transcription factors were predicted in the promoter region of genes involved in JH biosynthesis. The squares represent the predicted CREs. ATG represents the translation initiation codon.
